# Recent Insights into Neutrophil Extracellular Traps in Cardiovascular Diseases

**DOI:** 10.3390/jcm11226662

**Published:** 2022-11-10

**Authors:** Yuan Dong, Yuejie Zhang, Xuanyi Yang, Cen Yan, Yingmei Feng

**Affiliations:** Department of Science and Technology, Beijing Youan Hospital, Capital Medical University, Beijing 100069, China

**Keywords:** cardiovascular disease, atherosclerosis, neutrophil extracellular traps, NETosis, non-infectious inflammation

## Abstract

Neutrophils are primary effector cells of the innate immune system. Emerging evidence has consistently shown that activated neutrophils produce and release neutrophil extracellular traps (NETs) that play roles in immunity and non-infectious diseases. NETs are composed of DNA and proteins and serve as a structural platform for pathogen sequestration and degradation. In contrast to their protective role during pathogenic infection, NETs are pathologically involved in cardiovascular disease (CVD). In this review, we introduce the formation, release, and clearance of NETs and the regulatory mechanisms of NETs formation, followed by an overview of the clinical evidence for the involvement of NETs in CVD. Because atherosclerosis is a fundamental part of the pathogenesis of CVD, we chose to focus on the mechanisms by which NETs promote endothelial cell damage and collaborate with macrophages and platelets to accelerate plaque progression and thrombosis. Finally, we present options for clinical intervention to inhibit NETs production and release in the treatment of CVD. In conclusion, this review integrates the latest findings and provides new insights into NETs, which represent a novel biomarker and therapeutic target in clinical practice.

## 1. Introduction 

Neutrophils are key regulators that link the innate and adaptive immune responses. They originate exclusively from hematopoietic stem cells in bone marrow. The interaction between C-X-C chemokine receptor (CXCR)4, which is expressed on mature neutrophils, and C-X-C motif chemokine ligand (CXCL)12, which is secreted by stromal cells, maintain neutrophils retention in the bone marrow. Disruption of CXCR4/CXCL12 signaling leads to neutrophil mobilization from the bone marrow niche into the blood [1,2,3]. Dead Neutrophils are eliminated by phagocytes for clearance. In aged neutrophils, CXCR4 expression is increased to enhance cell return to bone marrow and cleared by macrophages [4]. As the most abundant type of white blood cells, neutrophils possess multiple antimicrobial properties, including release of preformed granules, production of reactive oxygen species (ROS) and nitrogen species, phagocytosis, and formation of neutrophil extracellular traps (NETs) for pathogen clearance.

Apart from immune defense, neutrophils are implicated in a wide spectrum of diseases including cardiovascular disease (CVD). As the underlying cause of CVD, atherosclerosis is initiated by the damage of vascular endothelial cells. Neutrophils are the first line of inflammatory cells to be activated and recruited to damaged endothelial cells. After homing to the lesion site, they produce ROS and secrete cathepsin G and proteases to degrade extracellular matrix (ECM), all of which trigger endothelial cell injury and activation. All these events further enhance inflammatory cell infiltration and low-density lipoprotein (LDL) penetration. Furthermore, neutrophils produce myeloperoxidase (MPO) to exaggerate LDL oxidation and foam cell formation, as well as NETs, which stimulate enhanced inflammatory cytokine production by macrophages and plasmacytoid dendritic cells. The toxic components in NETs are detrimental for smooth muscle cell survival. The death of smooth muscle cells and foam cells, together with the degradation of ECM, results in thinning of the fibrous cap and the formation of rupture-prone vulnerable plaques [5,6]. Despite the fact that neutrophils have short lifespans of about 6–8 h in circulation [7], the released NETs can be sustained for several days to carry out the prolonged function of neutrophils. In the review, we focus on how NETs are implicated in cardiovascular diseases.

## 2. NET Formation in Neutrophil Activation and Death

NETs were discovered in 2004 and originally considered as a new form of cell death that was termed NETosis [8]. Subsequent research illustrated that activated neutrophils release NETs to clear pathogens and increase inflammation [9]. The basic NET structure is a complex comprising modified chromatin, histones, and proteins from neutrophils such like elastase and myeloperoxidase. NETs are built to capture bacterial, viral, and fungal pathogens, and release proteins that eliminate such pathogens [9], thereby amplifying neutrophil function.

Indeed, increasing evidence suggests that NETs are produced by dying or activated neutrophils in nature [8,10,11]. By staining extracellular DNA with a cell-impermeable dye, DNA extruded from neutrophils was detected by fluorometer after 10 min phorbol-12-myristate-13-acetate (PMA) stimulation [8]. When PMA-treated neutrophils were followed up for 60 min, cell death was initiated as evidenced by loosening nuclei lobules, chromatin de-condensation, and dilation between the inner and outer nuclear membrane space under transmission electron microscopy and confocal fluorescence microscopy. By 180 min of PMA stimulation, the nuclear envelope was disintegrated into small vesicles and granules were diminished in neutrophils. To visualize NETs formation during death, PMA-treated neutrophils were labeled with calcein blue for viable cells, Annexin V for apoptotic cells, and antibodies against histone 2A, histone 2B, DNA, or elastase for NETs signature. NETs emerged in Annexin V^+^ calcein blue-apoptotic neutrophils [10]. This study clearly explicated that NETs formation and release are the early responses of activated neutrophils and are maintained from the beginning of activation till death.

It is not fully understood how NETs are cleared, nor do we know what factors are involved in regulating NET clearance. Theoretically, chromatin and proteins in NETs could be degraded by DNase and protease enzymes, respectively. The dynamics of NET clearance are influenced by many factors and disease conditions. Impaired NET clearance has been reported in different autoimmune disorders, including systemic lupus, gout, and antiphospholipid syndrome [12].

## 3. Regulation of NETs Formation and Clearance

It is well known that both extrinsic and intrinsic stimuli can induce the formation of NETs. Foreign pathogens are the main type of extrinsic stimulus, while the many intrinsic stimuli include lipopolysaccharide, cholesterol, cholesterol, and hydrogen peroxide [13,14]. Despite different stimuli promoting NETs formation via diverse signaling pathways, oxidative stress is the common mediator downstream of all the stimuli. The key regulators in NETs formation are summarized below.

### 3.1. PAD4 in NETs Formation

Protein arginine deiminases (PADs) are a family of Ca^2+^-dependent enzymes. They hydrolyze peptidyl arginine into peptidyl citrulline on histones, resulting in the loss of positive charge on the protein. By doing so, the target proteins undergo conformational change at the post-translational level with altered functions [15]. Five PAD family members have been identified by far with different distribution patterns in tissues. PAD1-3 are mainly expressed in human epidermis [16]. PAD6 is expressed mainly in germ cells [17]. PAD4 is mainly expressed in immune cells [15]. Nuclear PAD4 plays the key role in NETs formation. Citrullination in histone proteins by PAD4 weakens the interaction of these proteins with negatively charged DNA, leading to the release of the unwound, free strands of DNA for NETs formation. In PAD4-deficient mice, bleomycin-induced NET production and its associated pulmonary inflammation and fibrosis were significantly reduced [18]. By contrast, overexpression of PAD4 elevated NET production and promoted a more severe breakdown of the blood–brain barrier in a murine model of stroke [19].

### 3.2. NAPDH Oxidase-Dependent Pathways in NETs Formation

NAPDH oxidase is a multiprotein complex containing membrane and cytosolic components. The membrane component includes gp91phox and p22phox subunits and the cytosolic component comprises p47phox, p67phox, and p40phox. Upon stimulation by bacteria, free fatty acid [20], hydrogen peroxide [21], PMA [22], etc., cytosolic components are translocated to membrane components for activation of NAPDH oxidase [23]. The active NAPDH oxidase converts oxygen to superoxide for H_2_O_2_, MPO, and ROS production and the subsequent activation of nuclear PAD4 [24,25]. In patients with chronic granulomatous disease, mutations of NAPDH oxidase subunits are the cause of chronic granulomatous disease (CGD) in humans [26]. CGD patients could not produce ROS or NETs and are featured with severe immunodeficiency and high susceptibility to *Aspergillus* infection. NAPDH oxidase gene transfer using the SF71gp91phox vector restored NAPDH oxidase function and NETs formation, which was accompanied by rapid recovery of aspergillosis infection in CGD patients [26].

Different stimuli could promote NAPDH oxidase activation via different signaling pathways. For instance, acting through TLR2, TLR4, oxLDLactivated Protein kinase C (PKC)-Interleukin-1 receptor associated kinase (IRAKs)-mitogen-activated protein kinase (MAPK) pathway, leading to NAPDH oxidase activation, ROS production, and NETs formation in cultured neutrophils. Inhibition of PKC, IRAKs, or MAPK abrogated NAPDH oxidase activation and NETs formation in neutrophils treated with oxLDL [27]. Free fatty acids such like oleic acid, linoleic acid, and palmitic acid could trigger NETs formation and release. Exposure of oleic acid to cultured neutrophils induced ERK, 38MAPK and JNK phosphorylation together with NAPDH oxidase activation. Suppression of either factor above using its specific inhibitor could jeopardize NETs formation induced by oleic acid [20].

### 3.3. NAPDH Oxidase-Independent Pathways in NETs Formation

NETs production also occurs via an NADPH oxidase-independent mechanism. For example, the addition of uric acid promoted ROS production and NET formation in cultivated neutrophils isolated from human blood [28] without activation of NADPH oxidase. Neutrophil elastase and MPO were potent triggers for NETs formation, which were downstream of NADPH oxidase dependent and independent pathways. In vitro, exposure of *Candida albicans* to purified human neutrophils promoted translocation of neutrophil elastase and MPO into the nucleus where they synergistically degraded specific histones and decondensed DNA, leading to NET formation [29]. When cultivated neutrophils were exposed to PMA or A23187, calcium ionophores, the results of immunofluorescence assay revealed that A23187 triggered a faster NETs formation than PMA. When dissected further, the calcium-activated small conductance potassium (SK) channel member SK3 was activated by A23187, leading to increased Ca^2+^ influx and mitochondrial ROS production [30].

### 3.4. DNase I in NETs Clearance

Deoxyribonuclease I (DNase I) is an endonuclease that preferentially recognizes and cleaves double-stranded DNA in a Ca^2+^-dependent manner. Thus, it becomes one of the most potent NET scavengers [31]. A study by Brinkmann et al. was the first to demonstrate that NETs could be degraded by the addition of DNase I in vitro [8]. Dhawan et al. later demonstrated that correction of a defective DNase response by exogenous supplementation of DNase I in *ApoE*^−/−^ mice with advanced atherosclerosis resulted in a decrease in plaque NET content and significant plaque remodeling, with a decreased area of plaque necrosis and increased collagen content [32]. These data suggest that the clearance of NETs mediated by endonucleases plays an important role in preventing the advanced atherosclerotic plaque progression. 

**Figure 1:**
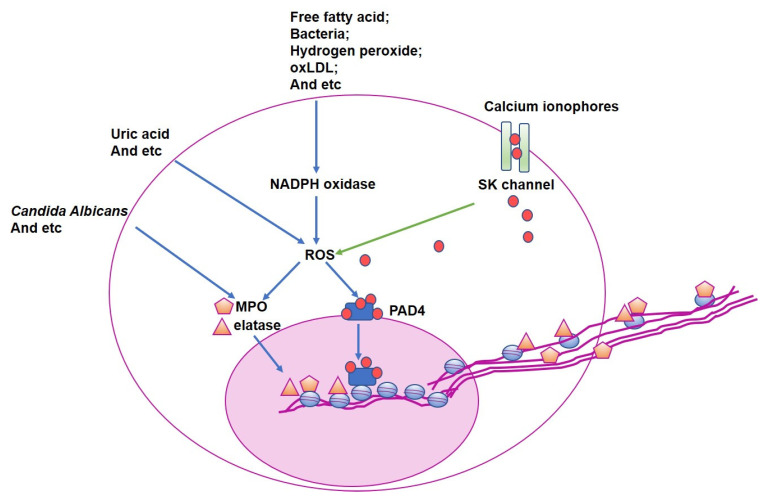
The mechanisms underlying NETs formation. Despite different stimuli promoting NETs formation via diverse signaling pathways, oxidative stress is the common mediator downstream of all the stimuli. Downstream of NADPH oxidase-dependent and independent pathways, reactive oxygen species (ROS) production is elevated that activates PAD4 and enhances MPO and elastase production. Citrullination in histone proteins by PAD4 weakens the interaction of these proteins with negatively charged DNA, leading to the release of the unwound, free strands of DNA for NETs formation. MPO and elastase translocate into the nucleus and degrade specific histones on DNA. Ultimately, NETs are formed and released from neutrophils. oxLDL, oxidized low-density lipoprotein; PAD4, peptidyl arginine deiminase 4; ROS, reactive oxygen species; MPO, myeloperoxidase.

The mechanisms underlying NETs formation are summarized in Figure 1.

## 4. Clinical Evidence of NETs in CVD

Evidence accumulating over decades has consistently shown that NETs represent a crucial component of host defense but also participate in many non-infectious diseases. Hereafter, we focus on the roles of NETs in CVD.

### 4.1. NETs Levels Related to Major Adverse Cardiovascular Events (MACE)

The relationship between NETs and CVD was assessed in a prospective study involving 282 patients with suspected CVD. The degree of coronary stenosis was determined by coronary computed tomography angiography. The hallmark components of NETs—double-stranded DNA, nucleosomes, citrullinated histone H4 and MPO-DNA complexes—were quantified by an enzyme-linked immunosorbent assay. The circulating levels of double-stranded DNA and nucleosomes were much higher in patients with severe stenosis and calcification in the coronary arteries than in those with mild or moderate stenosis. Using multivariate-adjusted analysis, baseline levels of NETs were positively associated with major adverse cardiovascular events (MACE) after 54 days of follow-up [33].

In a case-control study, 100 patients who experienced MACE were matched with 200 controls. By multivariate logistical analysis, the odds ratio for experiencing MACE was 1.94-fold for a composite of platelet count, soluble P-selectin, and all NET markers after 1 year of follow-up [34]. In line with that, enriched NETs and activated platelets were detected in the thrombi or plasma samples of patients with ST-elevated MI [35], acute coronary syndrome [36,37], or stroke [38].

### 4.2. DNase I SNP in Cardiovascular Mortality

Q222R is a type of DNase I single nucleotide polymorphism. Homozygous Q222R subjects manifest impaired DNase I function [39]. When patients with or without ST-segment elevation MI were genotyped for DNase I single nucleotide polymorphisms, Q222R was found in both groups at similar frequencies, both of which was associated with reduced DNase I activity. When DNase activity, double-stranded DNA, and citrullinated histone H3 were determined in a coronary culprit site and peripheral plasma, NETs burden was accumulated in the coronary culprit site rather than peripheral blood among all study patients, quantified by the amount of double-strand DNA and histone H3, and DNase I activity. Multivariable Cox regression analysis revealed that the hazard ratio of cardiovascular and all-cause mortality was independently related to 2.02-fold and 2.01-fold increases, respectively, in acute MI patients with homozygous Q222R variants compared with non-homozygous ones [39].

### 4.3. Histological Detection of NETs in Atherosclerotic Plaque

Moreover, histological and immunohistochemical staining detected the presence of NETs in coronary arterial thrombi, which was associated with extracellular iron and erythrocyte fragments in patients with ST-elevated MI [40]. Using immunostaining for neutrophil cell surface marker CD177 and elastase, NETs were identified by colocalization of neutrophil elastase and fluorescent 4′,6-diamidino-2-phenylindole-stained DNA in the luminal area [41]. These findings revealed that the cross-talk between NETs and different cells is crucial, and can determine the fate of plaque progression.

Evidence of NET participation in CVD is summarized in Table 1.

## 5. Evidence from Basic Research

The first study that demonstrated the presence of NETs in atherosclerotic plaque was traced back to 2012. The effects of neutrophils on atherosclerosis were studied in *ApoE*^−/−^ knockout mice expressing green fluorescent protein under the control of lysosome M. After 4–6 weeks on a western diet, the mice were injected with liposomes containing clodronate to deplete monocytes and then received carotid artery ligation. Immediately after ligation, neutrophils were visualized by intravenous injection of propidium iodide to label DNA. Using the two-photon microscopic intravital approach, neutrophils that had adhered to the lesion site and released DNA were detected. Conversely, in mice on a chow diet, neutrophils neither adhered to the injury site nor released DNA [41]. Furthermore, NETs were identified in the lipid-rich area by the staining of citrullinated histone 3 in the plaque of *ApoE*^−/−^ mice on 8 weeks of a high-fat diet [42]. Accordingly, plaque size was reduced 3-fold in mice deficient for *ApoE*/neutrophil elastase/proteinase 3 compared with that in *ApoE*^−/−^ mice after 8 weeks of a high-fat diet, suggesting the crucial role of NETs in atherosclerotic progression [42].

### 5.1. NETs and Endothelial Cells

As described above, atherosclerosis starts with damage to vascular endothelial cells. It is well established that plaque arises from arterial branches and curvatures where blood flow is disturbed. In response to wall shear stress, endothelial cells are activated to express a panel of adhesion molecules that elicit inflammation [43,44]. Apart from shear stress, NETs also act as a mediator for endothelial cell activation. When neutrophils isolated from healthy subjects were exposed to plasma collected from patients with essential hypertension, neutrophils became activated by factors released by platelets, leading to NETs formation [45]. Exposure of human aortic endothelial cells to NETs from diseased neutrophils modified the phenotype of these endothelial cells toward increased expression of vascular cell adhesion molecule-1, intercellular cell adhesion molecule-1, and collagen [45]. Likewise, systemic lupus erythematosus (SLE) is an independent risk factor of endothelial dysfunction and premature CVD. NETs isolated from SLE patients were enriched for MMP-2 compared with NETs from healthy controls. Moreover, NETs from SLE patients triggered reduced vasorelaxation but increased apoptosis of cultured murine endothelial cells compared with NETs from control subjects [46].

In line with in vitro findings, pathological effects of NETs on endothelial function have also been shown in vivo. Mice with flow-induced superficial erosion on the left carotid artery exhibited endothelial cell denudation that was associated with neutrophil adhesion to the lesion, as observed by Evans blue staining. When nanoparticles containing collagen IV and a PAD4 inhibitor were injected, the citrullinated histone-positive staining area within the neointima was reduced, indicating specific inhibition of NET formation. Consequently, the prohibition of NET formation in neutrophils by the PAD4 inhibitor promoted endothelial integrity and function [47].

### 5.2. NETs and Macrophages

Under hypercholesterolemia, modified LDL primes the formation of foam cell from macrophages and simultaneously stimulates the production of NETs by neutrophils [42]. IL-8, also known as CXCL8, behaves similarly to modified LDL and is the most intensively studied pro-inflammatory chemokine. In vitro, THP-1 macrophages stimulated with NET-containing plasma showed increased CXCL8 production and secretion. Acting through CXCR2, CXCL8 induced NET formation from neutrophils downstream of the Toll-like receptor (TLR)4-TLR9/nuclear factor-κB signaling pathway. Blockage of CXCR2 by intravenous injection of CXCR2 antibody abolished NET formation and plaque progression [48]. Secretion of the cytokine IL-1β by macrophages is a major driver of pathogenesis in atherosclerosis. In a study of *ApoE^−/−^* mice, NETs were found to prime macrophages for production of IL-1β and IL-6, resulting in the activation of a T_h_ 17 cell response, which amplified immune cell recruitment into atherosclerotic lesions [42].

Recently, an interesting study was performed to further dissect how NETs modify the macrophage phenotype in plaque. LDL receptor knockout (*LDLr*^−/−^) mice were placed on a western diet for 16 weeks. CD68^+^ macrophages in NET-positive and NET-negative areas in plaques were isolated using laser capture microscopy and subjected to RNA sequencing. Transcriptomic profiling analysis revealed elevated glycolysis and inflammasome in CD68^+^ macrophages taken from NET-positive areas compared with those from NET-negative areas. At week 17, some of the mice were placed back on a chow diet and received DNase I injections to degrade NETs for another 4 weeks. Comparable plaque regression was found in the mice that were returned to a chow diet, with or without DNase I administration. Another group of the *LDLr*^−/−^ mice that received a 16-week high-fat diet followed by a 4-week chow diet were injected with streptozotocin to induce diabetes in combination with the DNase 1 injection. Compared with non-diabetic controls, the DNase I treatment attenuated macrophages in plaque [49].

Taken together, the evidence strongly suggests that NETs are an important mediator between neutrophils and macrophages in atherosclerotic progression.

### 5.3. NETs and Platelets

Like macrophages, neutrophils engage in an intimate collaboration with platelets in atherosclerosis, in which NETs act as the central mediator. The role of DNase I-mediated degradation of NETs has also been shown in vivo in patients with acute thrombotic microangiopathies. Reduced plasma DNase I activity may lead to the persistence of pro-thrombotic NETs and thus promote microvascular thrombosis in TMA patients [50].

One of the most striking neutrophil–platelet interactions for NET formation is via P-selectin expressed on circulating platelets and P-selectin glycoprotein ligand 1 (PSGL-1) expressed on neutrophils [51]. Upon adhesion, PSGL-1 directly interacts with P-selectin, which stimulates elastase and cathepsin G production in the neutrophils to promote ECM degradation, NET formation, and propagated platelet activation [51,52,53]. As a negative feedback mechanism, elastase and cathepsin G also cleave PSGL-1 at the N-terminus to interrupt the P-selectin/PSGL-1 interaction [52]. Overall, the P-selection/PSGL-1 dependent recruitment of neutrophils to atherosclerotic plaque promotes neutrophil infiltration and subsequent destabilization of the plaque. A consequence of atheroprogression, following erosion and rupture of the plaque, is the initiation of thrombosis. In this condition, activated platelets secrete HMGB1, which self-stimulates platelets via the TLR4-myeloid differentiation primary response 88 (Myd88) axis and promotes NETosis through the receptor for advanced glycation end-products (RAGE) on neutrophils, thereby sustaining platelet activation and clot stability [54,55].

Additionally, a recent study showed that neutrophilic α9β1 promoted platelet activation and mutually enhanced NETosis and secretion of cathepsin G, thereby maintaining recruitment of arterial neutrophils [56,57]. Blocking platelet-induced NETosis by pharmacological or genetic ablation of PAD4 attenuated arterial thrombus formation [58]. Upon neutrophil activation, secreted NETosis promoted platelet activation and aggregation via complement C3 activation and subsequent platelet signaling through the anaphylatoxin receptor C3aR and downstream RAP1B [59]. Thus, C3aR deficiency reduced platelet activation and attenuated arterial thrombosis [60]. The mechanisms of these types of interactions between neutrophils and platelets are worth further investigation.

**Figure 2:**
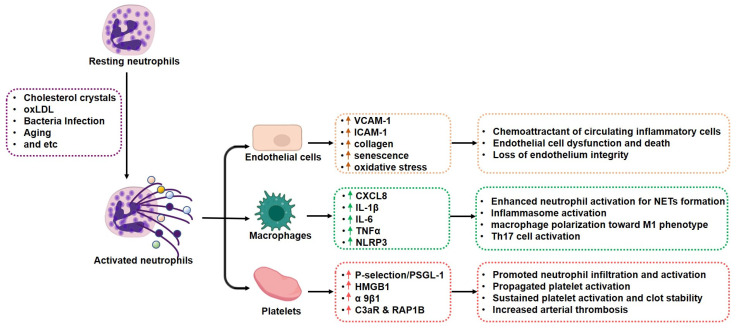
Cross-talk among neutrophil extracellular traps and cells in atherosclerotic plaque. Under intrinsic and extrinsic stimuli, NETs are formed mainly by two ways: NETosis from death neutrophils and vital NETs from activated neutrophils. Despite different forms, NETs are composed with modified chromatin, histones, and proteins from foreign pathogens, and granules and cytoplasm from neutrophils. The proteins in NETs mediate cell–cell communications. Upon adhesion to activated endothelial cells in the plaque, NETs promote adhesion molecular expression in endothelial cells to recruit circulating inflammatory cells into plaque. NETs stimulate macrophages to produce a series of inflammatory cytokines and polarization toward the M1 phenotype. NETs maintain platelet activation and thrombus formation. All the factors contribute to atherosclerotic plaque progression. The colorful balls released by neutrophils indicate different proteins.

The landscape of the cross-talk between NETs and cells involved in atherosclerotic progression is illustrated in Figure 2.

## 6. Interventional Strategies on NETs

### 6.1. PAD4 Inhibitors

The cysteine residue 645 in the active site of PAD4 covalently binds to its substrate benzoyl-arginine amide to catalyze out citrullination of arginine residues. Based on that, a series of pan-PAD inhibitors have been developed to suppress PAD4 enzyme activity. Chlor-amidine is an irreversible PADs inhibitor via covalent modification of the conserved cysteine residue in the active site of PADs [61]; GSK199 and GSK484 are reversible PAD inhibitors with high selectivity for PAD4. They are mimetic peptides of PAD4 substrate and competitively bind to PAD4. Crystal structures confirmed the binding of GSK199 and GSK484 to the PAD4 active site, part of which is rearranged to form a β-hairpin. All these compounds have shown to inactivate PAD4 and prohibit NETs formation in vitro cultured neutrophils [62].

### 6.2. Anticoagulants

Activated platelets release factors including high-mobility group box-1 (HMBG-1), platelet factor 4 (PF4), and CCL5 (RANTES) that promote neutrophils activation and NETs formation. Anticoagulants such as aspirin and heparin suppress platelet activation and block interaction between platelet and neutrophils. In the murine model of endotoxin-triggered acute lung injury, pretreatment of mice with aspirin circumvented platelet activation and blocked the interaction of platelets and neutrophils, leading to decreased intravascular NET formation and a reduced degree of lung injury [63].

### 6.3. NADPH Oxidase Inhibitor

Diphenyleneiodonium chloride is as a hypoglycemic agent able to block gluconeogenesis and respiration. It binds to the heme group of NAPDH oxidase and inhibits its enzyme activity and ROS production. In the murine model of liver transplantation, administration of *diphenyleneiodonium chloride* suppressed the NAPDH oxidase/ROS/PAD4 signaling pathway and rendered NETs production, all of which attenuated liver injury upon transplantation [64].

### 6.4. Antibiotics

Several antibiotics have been demonstrated to inhibit NETs formation. When freshly isolated human neutrophils were stimulated with PMA in the presence of gentamicin, azithromycin and chloramphenicol, NETs formation was substantially reduced [65,66].

### 6.5. Statins

Statins are the first-line pharmacological agents for the treatment of hypercholesterolemia in the primary and secondary prevention of atherosclerotic CVD. Radbakhsh et al. reported that simvastatin and trimetazidine had a NET-lowering effect that was likely related to the reduction in expression of IL-1β (a major driver of atherogenesis). They also reported that treatment of human neutrophils and HL-60 cells with cholesterol and methyl-β-cyclodextrin induced NETosis, while treatment with atorvastatin attenuated the cholesterol-induced NETosis [67].

### 6.6. Colchicine

Colchicine is a tricyclic, lipid-soluble alkaloid extracted from members of the lily family. It inhibits microtube polymerization and, therefore, prohibits inflammatory cell adhesion and recruitment. Particularly, it has been shown to suppress superoxide production and inflammasome activation, as well as NET release in neutrophils [68]. By far, it is the oldest known treatment for gout and has become a standard-of-care therapy for pericarditis [69]. Clinical trials have shown that administration of colchicine decreased the occurrence of MACE in patients with acute and chronic coronary syndrome [70]. Ongoing trials evaluating the effects of colchicine on atherosclerosis are underway [69].

## 7. Conclusions and Perspectives

Evidence is accumulating that NETs are involved in the pathogenesis of various types of non-infectious inflammation. Beyond the release of DNA, NETs contain proteins that act as core components, helping neutrophils to carry out their inflammatory destiny. Furthermore, the NET structure itself functions as a scaffold where different types of cells can be recruited to orchestrate the inflammatory cascade. By these means, NETs contribute to endothelial damage, platelet activation, thrombosis, and ischemia/reperfusion injury, making NETs a novel target in the treatment of CVD including atherosclerosis.

Despite potential strategies having been proposed and tested to block NETs formation, the off-targets of the drugs interfere with the net outcomes. For instance, pan-PAD inhibitors also bind to other PAD members in the body and modify the function in the tissues outside the lesion of interest. DNase I could digest not only NETs but also other double-strand DNA. Some antibiotics could suppress NETs formation, whereas others do not. As described above, diphenyleneiodonium chloride could prohibit a panel of enzyme activity including NAPDH oxidase. Therefore, how to avoid non-specific inhibition of enzyme activity is the next step for optimization of this compound. Do the antibiotics that inhibit NETs formation share any common biological, chemical, or physical features? How can we interpret whether any antibiotics could act on NETs formation? They are open questions to be investigated to control NETs formation more precisely, accurately, and efficiently. Defining the molecular pathways that are related to NETs will provide insight into potential therapeutic targets.

## Figures and Tables

**Table 1:** Summary of the roles of neutrophil extracellular traps (NETs) in cardiovascular diseases.

First Author	Type of Disease	Number of Patients	Main Findings
Megens et al. [41]	Atherosclerosis	Not mentioned	Plaque was dissected by endarterectomy for histological analysis. By performing immunostaining for neutrophil cell surface marker CD177 and elastase, NETs were identified by colocalization of neutrophil elastase and DAPI-stained DNA in the luminal area.
Mangold et al. [36]	ST-elevation acute coronary syndrome	111	Neutrophils and NETs were enriched in culprit lesion site thrombus. Thrombus NET burden positively related to infarct size, but culprit lesion site DNase activity negatively related to infarct size.
Stakos et al. [35]	STEMI	18	Thrombi from the infarct-related coronary artery contained activated platelets with NETs and active tissue factor.
Zhou et al. [38]	Stroke	55 stroke and 35 healthy controls	Plasma levels of NETs, activated platelets were higher in the carotid lesion than that in the aortic blood. NETs decorated with phosphatidylserine served as a platform for fibrotic factor deposition and thrombin and fibrin formation. In vitro, NETs promoted endothelial cell death.
Hofbauer et al. [39]	ST-segment elevation myocardial infarction (STEMI)	STEMI: 711 Controls: 1422	DNase 1 Q222R SNP was present in 64 (9.0%) STEMI patients, comparable to controls. Homozygous Q222R variant was independently associated with cardiovascular and all-cause mortality after STEMI.
Vaidya et al. [37]	Acute coronary syndrome (ACS)	60 (40 ACS and 20 stable angina pectosis	ACS patients were featured with increased NETs and MPO in the coronary sinus.
Hally et al. [34]	Acute myocardial infarction (AMI)	Case-control study: 100 cases vs. 200 controls	After one year of follow-up, the odds ratio for experiencing MACE was 1.94-fold for a composite of platelet count, soluble P-selectin, and all NETs markers.
Chilingaryan et al. [40]	STEMI	26	By performing histological and immunohistochemical staining, NETs were present in coronary arterial thrombi, which was associated with extracellular iron and erythrocyte fragments

DAPI = 4′,6-diamidino-2-phenylindole; STEMI = ST-segment elevation myocardial infarction; ACS = acute coronary syndrome; MACE = major adverse cardiovascular events.

## Abbreviations

CXCR	C-X-C chemokine receptor
CXCL	C-X-C motif chemokine ligand
CCL	chemokine C-C motif ligand
ROS	reactive oxygen species
NETs	neutrophil extracellular traps
CVD	cardiovascular disease
ECM	extracellular matrix
LDL	low-density lipoprotein
MPO	myeloperoxidase
PMA	phorbol-12-myristate-13-acetate
IL	interleukin
PBMC	peripheral blood mononuclear cell
MI	myocardial infarction
T_h_ 17	cells T helper 17 cells
PAD4	peptidyl arginine deiminase 4
HMGB1	high mobility group box 1
NASH	non-alcoholic steatohepatitis
MACE	major adverse cardiovascular events
SLE	systemic lupus erythematosus
*LDLr*^−/−^	LDL receptor knockout
TMAs	thrombotic microangiopathies
PSGL-1	P-selectin glycoprotein ligand 1
Myd88	myeloid differentiation primary response 88
RAGE	receptor for advanced glycation end-products
CGD	chronic granulomatous disease
IRAKs	Interleukin-1 receptor associated kinase
MAPK	mitogen-activated protein kinase
SK	calcium-activated small conductance potassium
TLR	toll-like receptor
PF4	platelet factor 4
